# Direct and Indirect Effects on Viral Translation and RNA Replication Are Required for AUF1 Restriction of Enterovirus Infections in Human Cells

**DOI:** 10.1128/mBio.01669-18

**Published:** 2018-09-04

**Authors:** Wendy Ullmer, Bert L. Semler

**Affiliations:** a Department of Microbiology and Molecular Genetics, School of Medicine, University of California, Irvine, California, USA; Virginia Polytechnic Institute and State University

**Keywords:** AUF1, IRES-mediated translation, RNA replication, coxsackievirus, enterovirus, host restriction, picornavirus, poliovirus

## Abstract

The cellular mRNA decay protein AUF1 acts as a restriction factor during infection by picornaviruses, including poliovirus, coxsackievirus, and human rhinovirus. AUF1 relocalizes from the nucleus to the cytoplasm during infection by these viruses due to the disruption of nucleocytoplasmic trafficking by viral proteinases. Previous studies have demonstrated that AUF1 binds to poliovirus and coxsackievirus B3 (CVB3) RNA during infection, with binding shown to occur within the internal ribosome entry site (IRES) of the 5′ noncoding region (NCR) or the 3′ NCR, respectively. Binding to different sites within the viral RNA suggests that AUF1 may negatively regulate infection by these viruses using different mechanisms. The work presented here addresses the mechanism of AUF1 inhibition of the replication of poliovirus and CVB3. We demonstrate that AUF1 knockdown in human cells results in increased viral translation, RNA synthesis, and virus production. AUF1 is shown to negatively regulate translation of a poliovirus and CVB3 IRES reporter RNA during infection but not in uninfected cells. We found that this inhibitory activity is not mediated through destabilization of viral genomic RNA; however, it does require virus-induced relocalization of AUF1 from the nucleus to the cytoplasm during the early phases of infection. Our findings suggest that AUF1 restriction of poliovirus and CVB3 replication uses a common mechanism through the viral IRES, which is distinct from the canonical role that AUF1 plays in regulated mRNA decay in uninfected host cells.

## Abstract (precis)

Picornaviruses primarily infect the gastrointestinal or upper respiratory tracts of humans and animals and may disseminate to tissues of the central nervous system, heart, skin, liver, or pancreas. Many common human pathogens belong to the *Picornaviridae* family, which includes viruses known to cause paralytic poliomyelitis (poliovirus); myocarditis (coxsackievirus B3 [CVB3]); the common cold (human rhinovirus [HRV]); and hand, foot, and mouth disease (enterovirus 71 [EV71]), among other illnesses. There are no specific treatments for infection, and vaccines exist for only two picornaviruses: poliovirus and hepatitis A virus. Given the worldwide distribution and prevalence of picornaviruses, it is important to gain insight into the host mechanisms used to restrict infection. Other than proteins involved in the innate immune response, few host factors have been identified that restrict picornavirus replication. The work presented here seeks to define the mechanism of action for the host restriction factor AUF1 during infection by poliovirus and CVB3.

## INTRODUCTION

The cytoplasmic life cycle of picornaviruses is dependent upon modification of many cellular processes and the repurposing of host proteins for the generation of progeny virions. As part of the alteration of the host cell landscape to promote virus replication, picornaviruses modify lipid metabolism and reorganize membrane architecture to form replication complexes, downregulate host cell transcription and translation to redirect cellular resources to favor viral replication, and disrupt nucleocytoplasmic trafficking to relocate nuclear proteins required for replication into the cytoplasm (1–4). Along with modification of cellular pathways to favor virus replication, picornaviruses must also disrupt processes that restrict virus replication. Triggered by double-stranded RNAs that form during viral RNA synthesis, the innate immune system is the best-described process that restricts virus replication, and as a result, picornaviruses have evolved numerous strategies to counteract this response (5, 6). Outside of the antiviral activity of the innate immune system, few host factors have been identified that restrict picornavirus replication. However, the dependence on cellular proteins to promote each step of the virus replication cycle provides opportunities for viral interactions with cellular restriction factors.

Upon release of a single-stranded, positive-sense RNA genome into infected cells, picornavirus proteins are translated by a cap-independent mechanism using an internal ribosome entry site (IRES) encoded within the 5′ noncoding region (NCR) of viral RNA. This structured region of RNA interacts with both canonical translation initiation factors and other host proteins known as IRES transacting factors (ITAFs) to recruit ribosomes to viral RNA (7–9). To date, a number of host proteins have been proposed to act as ITAFs for picornavirus translation, including the established ITAFs polypyrimidine tract-binding protein (PTB), poly(rC)-binding proteins 1 and 2 (PCBP1/2), upstream of N-ras (Unr), lupus La protein (La), nucleolin, and serine- and arginine-rich splicing factor 3 (SRSF3 or SRp20) (7, 9). In addition, three host proteins have been identified that restrict picornavirus translation: double-stranded RNA binding protein 76 (DRBP76), K homology-type RNA splicing regulatory protein (KHSRP, KSRP, or FBP2), and AU-rich element degradation factor 1 (AUF1 or hnRNP D).

DRBP76, KHSRP, and AUF1 were all identified in RNA affinity screens of the 5′ NCRs of different members of the *Enterovirus* genus of the *Picornaviridae* family. DRBP76, an isoform of interleukin enhancer binding factor 3 (ILF3), was found to bind the 5′ NCR of human rhinovirus 2 (HRV2) RNA and restrict infection in a cell-type-specific manner by negative regulation of the viral IRES (10, 11). KHSRP has been characterized as a negative ITAF for enterovirus 71 (EV71) following binding to multiple sites within its 5′ NCR (12). The ability of KHSRP to act as a negative ITAF is regulated by ubiquitination, which appears to enhance its ability to compete for binding to the EV71 IRES with a positive regulator of viral translation, far upstream element-binding protein 1 (FUBP1) (13). Of the identified restriction factors, AUF1 is the only protein shown to negatively regulate replication of several picornaviruses. Using knockdown or knockout mouse or human cell models, AUF1 has been shown to negatively regulate infection by poliovirus, coxsackievirus B3 (CVB3), HRV, and EV71 (14–17).

AUF1 is most often described as an mRNA decay protein that regulates the stability and translation of mRNAs following binding to sites within the 3′ NCR or introns of target transcripts. Four isoforms of AUF1 are generated through alternative pre-mRNA splicing and are named based on their apparent molecular weights: p37, p40, p42, and p45 (18). All four isoforms of AUF1 are predominantly nuclear proteins, but the smaller isoforms, p37 and p40, shuttle between the nucleus and cytoplasm (19). During infection by poliovirus, CVB3, HRV, or EV71, AUF1 was shown to relocalize to the cytoplasm following disruption of nucleocytoplasmic trafficking by viral proteinases (14–17, 20, 21). Additionally, AUF1 relocalizes to the cytoplasm during infection by encephalomyocarditis virus (EMCV), a nonhuman pathogen belonging to the *Cardiovirus* genus of *Picornaviridae*. However, AUF1 does not negatively regulate infection of this virus in a mouse cell model (15).

The role of AUF1 as a restriction factor during picornavirus infection has been primarily described as that of a negative regulator of viral translation. AUF1 has been shown to bind poliovirus, CVB3, and EV71 RNA during infection, suggesting that restriction occurs through interaction with viral RNA (16, 17, 22). For poliovirus, HRV, and EV71, binding has been shown to occur in the 5′ NCR, specifically within stem loops that make up the poliovirus and EV71 IRES (14, 16, 17). Consistent with these results, AUF1 has been shown to reduce *in vitro* translation of poliovirus RNA (14). Using bicistronic reporter assays, AUF1 was shown to negatively regulate EV71 IRES-driven translation, likely through competition with the positive ITAF, hnRNP A1 (17, 23, 24). Given its role in mRNA decay, AUF1 may also restrict picornavirus infection through degradation of viral RNA. AUF1 was found to bind to a reporter RNA harboring a CVB3 3′ NCR, and knockdown of AUF1 was shown to stabilize that RNA (16). These data suggest that AUF1 may regulate the stability of CVB3 RNA through binding of its 3′ NCR.

In the study described in this report, we investigated the mechanism by which AUF1 acts as a restriction factor during poliovirus or CVB3 infection of human cells. Following AUF1 knockdown, infection by poliovirus and CVB3 resulted in increased viral translation, RNA synthesis, and progeny virion production. Although AUF1 targets many cellular mRNAs by binding within the 3′ NCR (25), we found that AUF1 was able to restrict the replication of a mutant poliovirus lacking its 3′ NCR, demonstrating that restriction of poliovirus infection does not occur through binding to its 3′ NCR. Importantly, our data showed that AUF1 had no detectable effect on the stability of poliovirus or CVB3 RNA during infection. Using poliovirus and CVB3 5′ NCR reporter RNAs, we demonstrate that AUF1 negatively regulates both poliovirus and CVB3 IRES-driven translation during infection. These findings revealed that the effect of AUF1 on enterovirus RNA synthesis is, in part, indirect due to a reduction in the levels of viral proteins required for replication. Finally, we demonstrated that AUF1 had no effect on viral IRES-driven translation in uninfected cells, suggesting that relocalization of AUF1 from the nucleus to the cytoplasm during infection is a crucial requirement for its negative effects.

## RESULTS

### AUF1 negatively regulates replication of poliovirus and CVB3 in human cells.

**FIG 1:**
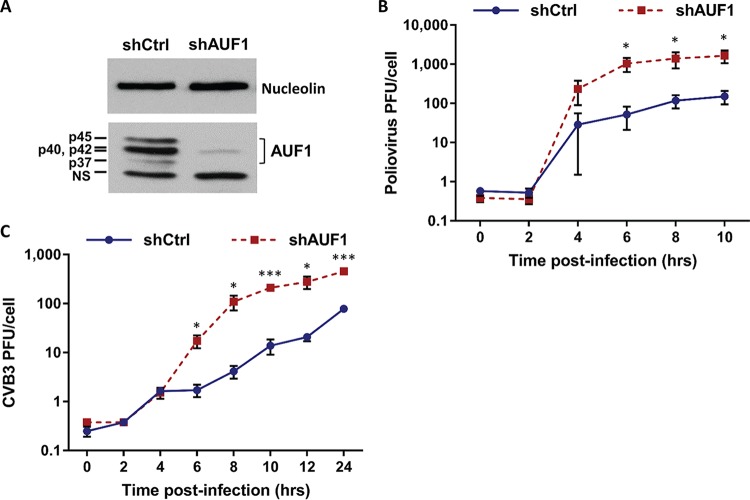
AUF1 knockdown enhances replication of poliovirus and CVB3 in HEK-293 cells. (A) Cell lysates were prepared from HEK-293 cells stably expressing control (shCtrl) or AUF1 (shAUF1) targeting shRNAs and analyzed for AUF1 protein expression by Western blotting. The four isoforms of AUF1 (p37, p40, p42, and p45) are labeled, and nucleolin was used as a loading control. NS, nonspecific band. (B and C) 293-shCtrl or -shAUF1 cells were infected with poliovirus at an MOI of 1 (B) or CVB3 at an MOI of 20 (C). Cells and supernatant were harvested at specified time points, and virus titer was determined by plaque assay on HeLa cells. Data represent the means from three individual experiments ± standard errors of the means (SEM). *, *P* < 0.05; **, *P* < 0.01; ***, *P* < 0.005.

AUF1 has previously been shown to negatively regulate replication of several picornaviruses. Poliovirus was found to replicate to higher titers in mouse embryonic fibroblasts (MEFs) genetically ablated for AUF1 (14). However, since mouse cells do not express the human poliovirus receptor (PVR), the effect of AUF1 on virus replication was measured following transfection of *in vitro*-transcribed or virion RNA into cells. The impact of AUF1 on poliovirus replication in human cells, the natural host for this virus, has not been measured. To assess the effect of AUF1 on replication of poliovirus in a human cell model, HEK-293 cells stably expressing a short hairpin RNA (shRNA) targeting all four isoforms of AUF1 (293-shAUF1) were generated. Expression of shAUF1 resulted in over a 90% reduction of AUF1 protein expression relative to control (293-shCtrl) cells (Fig. 1A). Following infection by poliovirus at a multiplicity of infection (MOI) of 1, an approximately 10-fold increase in poliovirus titer was observed by 6 h after infection of 293-shAUF1 cells (Fig. 1B). These results demonstrate that AUF1 restricts poliovirus replication during infection of a human cell line, similar to the observations made in mouse cells.

Knockout or knockdown of AUF1 in MEFs or HeLa cells, respectively, has been shown to increase replication of CVB3 (14, 16). To confirm that AUF1 also negatively regulates replication of CVB3 in HEK-293 cells, 293-shCtrl or -shAUF1 cells were infected with CVB3 at an MOI of 20. Consistent with previous results, an approximately 10-fold increase in CVB3 titer was observed in 293-shAUF1 cells by 8 h postinfection (Fig. 1C). These data demonstrate that AUF1 negatively regulates infection by the enteroviruses poliovirus and CVB3 in multiple cell types.

### Poliovirus and CVB3 RNA synthesis is negatively regulated by AUF1.

**FIG 2:**
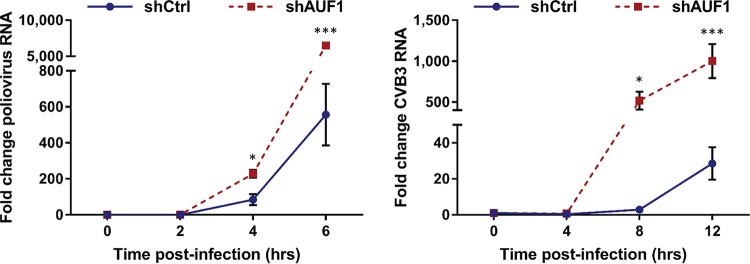
AUF1 inhibits poliovirus and CVB3 RNA synthesis. 293-shCtrl or -shAUF1 cells were infected with poliovirus (MOI of 1) or CVB3 (MOI of 20), and RNA was extracted at specified time points. Viral RNA synthesis relative to the 0-h time point for each cell line was analyzed by RT-qPCR using the ΔΔ*C*_*T*_ method. Data represent the means from three individual experiments ± standard errors of the means (SEM). *, *P* < 0.05; *** *P*, < 0.005.

The effect of AUF1 on picornavirus RNA synthesis has been measured for CVB3 and EV71. AUF1 knockdown was shown to increase synthesis of CVB3 RNA in HeLa cells, while there was no effect on EV71 RNA synthesis in the human glioblastoma cell line SF268 (16, 17). These apparently discordant results suggest that the negative impact of AUF1 on enterovirus replication may differentially affect viral RNA synthesis. To determine the effect of AUF1 on poliovirus and CVB3 RNA replication in HEK-293 cells, viral RNA was measured using quantitative reverse transcription-PCR (RT-qPCR). Following infection by either poliovirus or CVB3, a significant increase in viral RNA synthesis was observed in 293-shAUF1 cells (Fig. 2). The increase in viral RNA synthesis was detected by 4 h and 8 h postinfection for poliovirus and CVB3, respectively. These results demonstrate that AUF1 negative regulation of poliovirus and CVB3 infection results in decreased viral RNA synthesis. However, these results do not distinguish between a direct and an indirect effect on viral RNA synthesis since restriction of other steps in the replication cycle or destabilization of viral RNA may contribute to the decrease in viral RNA.

### AUF1 does not restrict poliovirus replication through its 3′ noncoding region or promote viral RNA decay.

**FIG 3:**
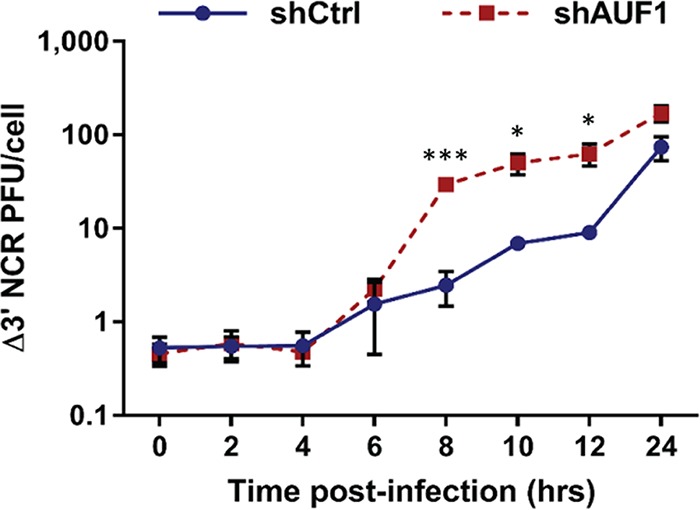
AUF1 does not negatively regulate poliovirus through its 3′ NCR. 293-shCtrl or -shAUF1 cells were infected with poliovirus lacking its 3′ NCR (Δ3′ NCR) at an MOI of 1. Cells and supernatant were harvested at specified time points, and virus titer was determined by plaque assay on HeLa cells. Data represent the means from three individual experiments ± standard errors of the means (SEM). *, *P* < 0.05; ***, *P* < 0.005.

AUF1 has been shown to bind the CVB3 3′ NCR using a 3′ NCR reporter construct expressed in HeLa cells. Based upon small interfering RNA (siRNA)-mediated knockdown of AUF1 in HeLa cells and an indirect measurement of RNA stability, Wong and colleagues concluded that AUF1 may restrict CVB3 replication by promoting viral RNA degradation analogous to its role in mRNA decay (16). To determine whether AUF1 negatively regulates poliovirus replication through binding of the 3′ NCR, a mutant poliovirus lacking the 3′ NCR (Δ3′ NCR) of its genomic RNA was utilized. Poliovirus Δ3′ NCR is an infectious virus but exhibits a replication impairment due to a reduction in positive-strand RNA synthesis (26, 27). Following infection by the Δ3′ NCR virus, an approximately 10-fold increase in virus titer was measured in 293-shAUF1 cells, similar to the effect of AUF1 knockdown on wild-type poliovirus (Fig. 3). As previously reported, there were overall reduced virus yields and a delay in replication of the Δ3′ NCR virus compared to wild type; however, the effect of AUF1 knockdown on replication of the Δ3′ NCR virus demonstrates that AUF1 does not negatively regulate poliovirus through its 3′ NCR. The effect of AUF1 on replication of CVB3 lacking its genomic 3′ NCR could not be assessed due to lack of viability of the CVB3 Δ3′ NCR virus (data not shown).

**FIG 4:**
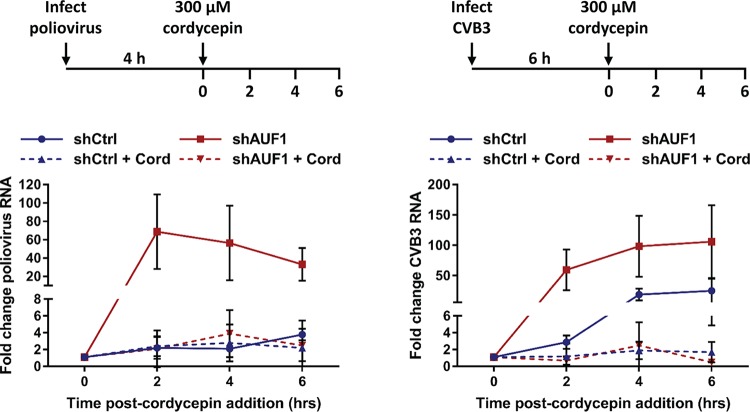
AUF1 does not promote poliovirus or CVB3 RNA decay. 293-shCtrl or -shAUF1 cells were infected with poliovirus (MOI of 1) or CVB3 (MOI of 20) and treated with vehicle (DMSO) or 300 µM cordycepin (Cord) at the indicated times postinfection. RNA was extracted at 0, 2, 4, or 6 h after cordycepin addition, and the fold change in viral RNA relative to the 0-h time point for each cell line was determined by RT-qPCR using the ΔΔ*C*_*T*_ method. Data represent the means from three or four individual experiments ± standard errors of the means (SEM).

Although the 3′ NCR of poliovirus genomic RNA is not involved in negative regulation of infection by AUF1, it is possible that AUF1 destabilizes viral RNA through interactions with RNA sequences outside the 3′ NCR. To directly measure the stability of poliovirus and CVB3 RNA during infection, viral RNA synthesis was inhibited using the adenosine analog cordycepin (3′-deoxyadenosine), which, when taken up by cells and phosphorylated to produce cordecypin triphosphate, acts as a chain terminator for RNA synthesis (28). Cordycepin has been previously shown to inhibit poliovirus and HRV14 RNA synthesis and is more effective at inhibiting RNA synthesis at later times of infection than the commonly used inhibitor guanidine hydrochloride (GuHCl). Cells were infected with poliovirus or CVB3, and cordycepin was added to the culture medium at 4 h or 6 h postinfection, respectively (Fig. 4). Viral RNA synthesis was inhibited at a time during infection when there is a detectable increase in viral RNA (Fig. 2) and when AUF1 has relocalized to the cytoplasm (16, 21). Upon inhibition of viral RNA synthesis, the stability of viral RNA was analyzed for the next 6 h by RT-qPCR. Consistent with our previous results, in both the untreated poliovirus- and CVB3-infected 293-shAUF1 cells, viral RNA was synthesized at higher levels than in 293-shCtrl cells. However, when viral RNA synthesis was inhibited by cordycepin, there was no measurable difference between viral RNA stability in the 293-shCtrl and -shAUF1 cells for either poliovirus or CVB3. These data demonstrate that AUF1 does not restrict infection by these viruses through destabilization of viral RNA.

### AUF1 inhibits poliovirus and CVB3 translation during infection.

**FIG 5:**
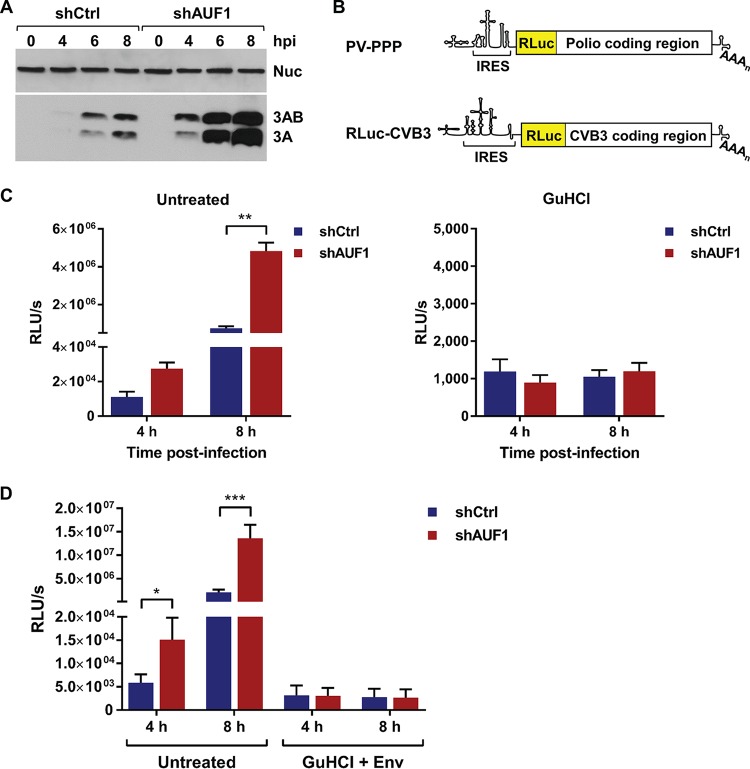
AUF1 restricts poliovirus and CVB3 translation during infection of HEK-293 cells. (A) Protein lysates were generated from 293-shCtrl or -shAUF1 cells infected with poliovirus (MOI of 1) at 0, 4, 6, and 8 h postinfection (hpi) and analyzed for viral 3A/3AB expression by Western blotting. Nucleolin was used as a loading control. (B) Schematic diagrams of PV-PPP (top) and RLuc-CVB3 (bottom) RNA. Both RNAs contain a full-length viral genome with the *Renilla* luciferase (RLuc) gene immediately following the 5′ NCR and a 3C cleavage site to liberate RLuc from the viral polyprotein. (C) 293-shCtrl or -shAUF1 cells were infected with PV-PPP with or without 5 mM guanidine hydrochloride (GuHCl) treatment during and after adsorption. Luciferase activity was measured at 4 and 8 hpi and represented as relative light units per second (RLU/s) normalized to cell count. The means from three individual experiments ± SEM are represented. (D) 293-shCtrl or -shAUF1 cells were infected with RLuc-CVB3 and treated with 5 mM GuHCl and 5 µg/ml enviroxime (Env) during and after adsorption. Luciferase activity was measured at 4 and 8 hpi and represented as relative light units per second (RLU/s) normalized to cell count. The means from three individual experiments ± SEM are represented. *, *P* < 0.05; **, *P* < 0.01; ***, *P* < 0.001.

The inhibitory effect of AUF1 on poliovirus translation was previously measured using *in vitro* translation assays (14). Given that cell-free assays are unable to recapitulate the nuclear-cytoplasmic partitioning of a host protein like AUF1, we turned to cell culture assays in the context of viral infection. Following infection of HEK-293 cells, viral protein accumulation was analyzed by Western blotting. Production of viral protein 3A, and its precursor 3AB, revealed a clear increase in poliovirus translation in 293-shAUF1 compared to 293-shCtrl cells by 4 h postinfection (Fig. 5A). Since the observed increase in viral translation may have been the result of increased viral RNA synthesis and subsequent translation of these progeny RNAs, *Renilla* luciferase-expressing poliovirus (PV-PPP) and CVB3 (RLuc-CVB3) were used to separate the effect of AUF1 on viral translation or RNA synthesis. (Fig. 5B). In untreated cells, quantification of luminescence represented translation of both input and newly synthesized viral RNAs. To separate the two processes, viral RNA synthesis was inhibited using either GuHCl for poliovirus or GuHCl combined with enviroxime (Env) for CVB3 (29–31). Inhibition of viral RNA synthesis allowed for the measurement of translation from input viral RNA only, without the contribution of newly synthesized RNAs. Cells treated with RNA synthesis inhibitors were treated both during and after adsorption to ensure complete inhibition of viral RNA synthesis. Following infection by PV-PPP or RLuc-CVB3, luciferase activity was measured at 4 h and 8 h postinfection. In untreated cells, luciferase activity was higher in 293-shAUF1 cells following PV-PPP or RLuc-CVB3 infection (Fig. 5C and D). Significantly, when viral RNA synthesis was inhibited following either PV-PPP or RLuc-CVB3 infection, there was no difference in luciferase activity measured from the 293-shCtrl or -shAUF1 cells (Fig. 5C and D). These data demonstrate that AUF1 negatively regulates poliovirus and CVB3 translation when infection is allowed to proceed normally but has no effect on translation of input viral RNA.

**FIG 6:**
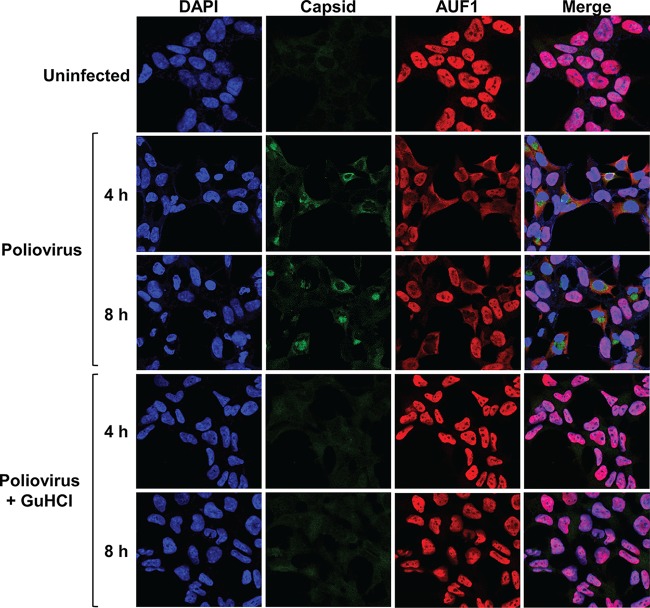
Inhibition of poliovirus RNA synthesis blocks nuclear-cytoplasmic relocalization of AUF1 during infection. HEK-293 cells were infected with poliovirus (MOI of 1) with or without 5 mM GuHCl treatment during and after adsorption. Cells were fixed at 4 and 8 h postinfection and analyzed for AUF1 (red) and poliovirus capsid (green) localization by immunofluorescence assay. Nuclei were counterstained with DAPI (blue). Cells were imaged using confocal microscopy.

Inhibition of viral RNA synthesis during infection leads to reduced accumulation of viral proteins in cells. Viral proteinase activity is required for the disruption of nucleocytoplasmic trafficking in infected cells, so low viral protein expression may prevent relocalization of nuclear proteins into the cytoplasm. Cytoplasmic relocalization of AUF1 occurs during poliovirus, CVB3, HRV, EV71, and EMCV infection and may contribute to its negative effect on virus replication (14, 15, 17, 20, 21). To determine whether inhibition of viral RNA synthesis prevents relocalization of AUF1, HEK-293 cells were infected with poliovirus with or without GuHCl treatment and AUF1 localization was analyzed by immunofluorescence assay (IFA) (Fig. 6). Following poliovirus infection of untreated cells, AUF1 clearly translocated from the nucleus to the cytoplasm of infected cells at 4 h and 8 h postinfection. However, in cells where viral RNA synthesis was inhibited, viral protein expression was not detected by IFA and AUF1 remained predominantly in the nucleus. Taken together, these results suggest that AUF1 does not negatively regulate translation of input poliovirus or CVB3 RNA but impacts viral translation at later stages of infection. Furthermore, the relocalization of AUF1 as an unintended consequence of the disruption of nucleocytoplasmic trafficking appears to be required for its negative effect on virus replication.

### AUF1 negatively regulates poliovirus and CVB3 IRES-driven translation.

**FIG 7:**
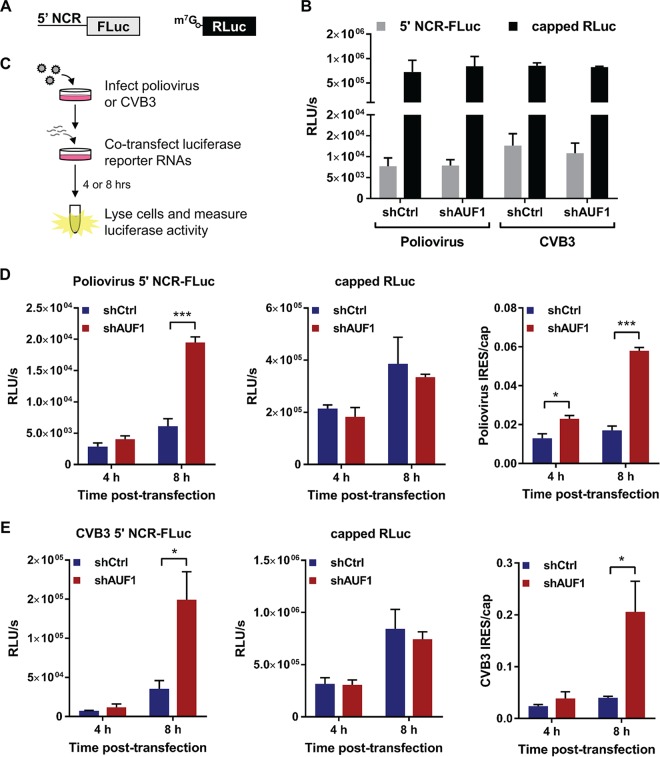
AUF1 inhibits poliovirus and CVB3 IRES-driven translation during infection. (A) Schematic of luciferase reporter RNAs. Viral IRES-driven translation was measured using *in vitro*-transcribed RNA encoding the poliovirus or CVB3 5′ noncoding region (5′ NCR) upstream of firefly luciferase (FLuc). Cap-dependent translation was measured using a *Renilla* luciferase (RLuc) construct *in vitro* transcribed in the presence of m^7^G(5′)ppp(5′)G cap analog. (B) Viral IRES- and cap-dependent translation measured in uninfected cells 8 h after cotransfection of 293-shCtrl or -shAUF1 cells. FLuc and RLuc activities were measured using a dual-luciferase assay and represented as relative light units per second (RLU/s) normalized to cell count. The means from three individual experiments ± SEM are represented. (C) Schematic of infection followed by cotransfection experiment. 293-shCtrl or -shAUF1 cells were infected with poliovirus (MOI of 1) or CVB3 (MOI of 20). Immediately following virus adsorption, cells were cotransfected with the 5′ NCR-FLuc and capped RLuc RNAs. At 4 h and 8 h posttransfection, cells were lysed and dual luciferase activity was measured. (D) 293-shCtrl or -shAUF1 cells were infected with poliovirus (MOI of 1) prior to cotransfection with luciferase reporter RNAs. FLuc and RLuc activities were measured at 4 h and 8 h posttransfection and represented as relative light units per second (RLU/s) normalized to cell count. Data from cotransfections were graphed separately due to differences in scale and represent the means from three individual experiments ± SEM. The ratios of IRES- to cap-dependent translation from these experiments are also presented. (E) 293-shCtrl or -shAUF1 cells were infected with CVB3 (MOI of 20) prior to cotransfection with luciferase reporter RNAs. Results are presented the same as in panel D. *, *P* < 0.05; ***, *P* < 0.001.

AUF1 has been previously shown to bind the 5′ NCR of poliovirus, HRV, and EV71 RNA (14, 17, 21). Evidence that AUF1 binds to stem-loop structures within the IRES of poliovirus and EV71 suggests that it may negatively regulate viral IRES-driven translation by acting as a negative ITAF, perhaps by competing with bona fide ITAFs for binding sites in the viral IRES (14, 17). The effect of AUF1 on poliovirus or CVB3 IRES-driven translation has not been measured in cells. To determine whether AUF1 negatively regulates the poliovirus or CVB3 IRES during infection, two different reporter RNAs were used. The first RNA harbors the 5′ NCR of poliovirus or CVB3 upstream of sequences encoding firefly luciferase (5′ NCR-FLuc), and the second encodes a control *Renilla* luciferase (RLuc) (Fig. 7A). The 5′ NCR-FLuc RNAs were *in vitro* transcribed in the absence of mRNA cap analog so that FLuc activity represented viral IRES-driven translation. The RLuc control RNA was *in vitro* transcribed in the presence of cap analog so that RLuc activity represented cellular cap-dependent translation. When uninfected cells were cotransfected with the 5′ NCR-FLuc and RLuc RNAs, there were no differences in either viral IRES- or cap-driven translation between the 293-shCtrl and -shAUF1 cells (Fig. 7B). To measure the effect of AUF1 on translation of the reporter RNAs during infection, cells were infected with poliovirus or CVB3 prior to cotransfection of the respective reporter RNAs (Fig. 7C). Following infection, an increase in both poliovirus and CVB3 IRES-driven translation was observed in the 293-shAUF1 cells (Fig. 7D and E). These results demonstrate that AUF1 negatively regulates poliovirus and CVB3 IRES-driven translation during infection but not in uninfected cells. The inability of AUF1 to negatively regulate viral IRES-driven translation in uninfected cells provides strong evidence that relocalization of AUF1 is required for its activity as a restriction factor during picornavirus infection.

## DISCUSSION

The work presented here provides new mechanistic insights into how AUF1 acts as a host restriction factor during enterovirus infections of human cells. AUF1 normally participates in mRNA stability, translation, and telomere maintenance in the host cell; however, AUF1 has been primarily described as an mRNA decay protein (32–34). Following binding to AU-, U-, or GU-rich regions of RNA in the 3′ NCR or introns of target transcripts, AUF1 promotes rapid deadenylation followed by decapping and degradation of the body of the mRNA (32, 35–38). It has been previously reported that AUF1 could bind to and promote the decay of a CVB3 3′ NCR reporter RNA in HeLa cells (16). These results, although indirect, were interpreted to show that AUF1 destabilizes viral RNA through binding to the 3′ NCR. Here, we used a mutant poliovirus lacking a 3′ NCR (Δ3′ NCR) in its genomic RNA to determine whether this region of RNA is required for AUF1 negative regulation of poliovirus infection. Knockdown of AUF1 resulted in increased replication of the Δ3′ NCR virus, similarly to wild-type poliovirus. These results demonstrate that the 3′ NCR is not involved in AUF1 negative regulation of poliovirus infection. The contribution of the CVB3 3′ NCR to AUF1 restriction of an authentic viral infection could not be analyzed due to the lack of viability of the CVB3 Δ3′ NCR virus (data not shown). Since AUF1 may regulate viral RNA stability by binding to regions of RNA outside the 3′ NCR, a direct measurement of viral RNA stability was performed. Use of an RNA chain terminator to inhibit viral RNA synthesis at middle times of infection revealed that poliovirus and CVB3 RNAs were equally stable in 293-shCtrl or -shAUF1 cells. These data demonstrate that AUF1 negative regulation of infection occurs by a mechanism distinct from its role in mRNA decay.

**FIG 8:**
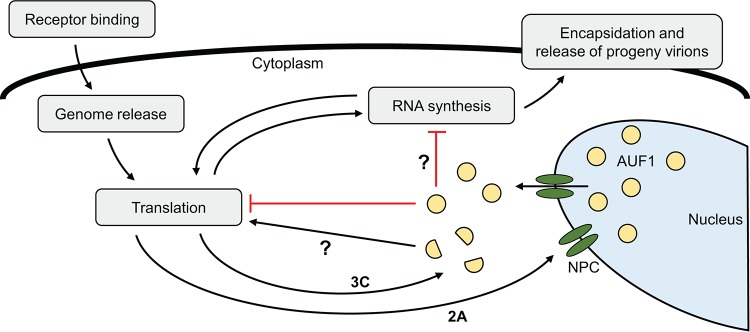
Proposed model for AUF1 negative regulation of poliovirus and CVB3 infection. Following poliovirus and CVB3 infection, disruption of the nuclear pore complex (NPC) by the viral 2A proteinase leads to relocalization of AUF1 from the nucleus to the cytoplasm. Once in the cytoplasm, AUF1 restricts infection through inhibition of viral translation by acting as a negative ITAF. Inhibition of viral translation leads to decreased viral RNA synthesis and generation of progeny virions. A direct inhibition of viral RNA synthesis may also contribute to the negative effect of AUF1 on infection, although the involvement of AUF1 in RNA replication complexes has yet to be analyzed. Poliovirus and CVB3 may defend against restriction by AUF1 through proteolytic cleavage of AUF1 by viral proteinases. AUF1 is cleaved by the 3C proteinase during infection, which may ameliorate its negative effect on viral translation and enhance virus replication.

The results presented here suggest that AUF1 negatively regulates poliovirus and CVB3 translation by acting as a negative ITAF during infection or by sequestering factors normally required for viral IRES-dependent translation. Viral IRES-driven translation was not affected by AUF1 in uninfected cells. Since AUF1 is predominantly a nucleus-resident protein in uninfected cells, or in infected cells treated with viral RNA synthesis inhibitors, low levels of AUF1 in the cytoplasm appear to be insufficient to negatively regulate translation initiated from the viral IRES. The disruption of nucleocytoplasmic trafficking during picornavirus infection results in a major relocalization of AUF1 into the cytoplasm, which has been demonstrated during poliovirus, CVB3, HRV, EV71, and EMCV infection. Once relocalized to the cytoplasm, AUF1 is present at sufficient levels to act as an inhibitor of viral translation (model shown in Fig. 8). It has been previously reported that AUF1 knockdown does not affect translation from the EMCV IRES in uninfected HeLa cells, but translation was not measured during infection, when AUF1 has been shown to relocalize in these cells (15, 39). It has also been reported that AUF1 knockdown in the human glioblastoma cell line SF268 resulted in increased EV71 IRES-driven translation following reporter RNA transfection. In contrast to the work presented here, these findings were determined in uninfected cells (17). The differences in the results presented here and by Lin and colleagues (17) may be attributed to variations in AUF1 subcellular localization between cell types or differences in experimental design. Luciferase activity was measured at 4 h and 8 h posttransfection in the work presented here and at 2 days posttransfection by Lin and colleagues (17). Given a longer incubation time, it may be possible that low levels of cytoplasmic AUF1 have a cumulative effect on viral IRES-driven translation.

A possible mechanism by which AUF1 negatively regulates picornavirus IRES-driven translation is through competitive binding to the IRES with positive ITAFs. Evidence for this mechanism was previously demonstrated using the EV71 5′ NCR, where AUF1 was shown to compete with another AU-rich element binding protein, hnRNP A1, for association with the IRES (17). This has not yet been shown for other picornaviruses negatively regulated by AUF1. AUF1 may also restrict picornavirus IRES-driven translation through indirect mechanisms. Following AUF1 knockdown, dysregulation of AUF1 target transcripts may contribute to increased IRES translation during infection. Additionally, the enhanced infection observed in AUF1 knockdown cells may drive IRES translation in a nonspecific manner. While indirect mechanisms may be possible, multiple reports of direct interactions between AUF1 and picornavirus RNA, specifically the 5′ NCR, support a direct mechanism for negative regulation.

Picornaviruses have evolved to extensively modify the host cell landscape to promote their replication and defend against restrictive cellular processes. Proteinases encoded by these viruses are employed to cleave cellular proteins, resulting in inhibition or modification of their activity. For example, the poliovirus, CVB3, and EV71 2A proteinases cleave MDA5 and MAVS, inhibiting viral RNA sensing and activation of the innate immune response (40). Picornaviruses may also inhibit restriction by AUF1 through proteolytic cleavage. AUF1 has been shown to be cleaved during poliovirus, CVB3, and HRV infection by the viral 3C/3CD proteinase, and preincubation of recombinant AUF1 with 3CD proteinase reduced its ability to bind to the poliovirus IRES *in vitro* (14, 16, 21). However, it is not yet clear whether the cleavage of AUF1 serves as a viral defense mechanism during infection (Fig. 8).

In this study, we have shown that knockdown of AUF1 resulted in increased replication of poliovirus and CVB3 in a human cell model. Enhanced infection of poliovirus and CVB3 resulted in increased viral RNA synthesis and translation in AUF1 knockdown cells. Our data demonstrated that the effect on viral RNA synthesis is largely indirect, due to the increased production of viral proteins required for replication in cells knocked down for AUF1 expression. In addition, we provided direct evidence that AUF1 does not exert its restrictive effects via alterations in RNA stability for either poliovirus or CVB3. Using 5′ NCR reporter RNAs, we showed that AUF1 negatively regulates poliovirus and CVB3 IRES-driven translation during infection but not in uninfected cells. These results suggest that the relocalization of AUF1 as an unintended consequence of the disruption of nucleocytoplasmic trafficking is required for its negative effect. Taken together, our findings reveal that AUF1 restricts viral translation subsequent to protein synthesis directed by input viral RNAs released from virions following uncoating and that poliovirus- and CVB3-encoded proteinases may serve as both the initiators of AUF1 antiviral defense activity and, ultimately, the destroyers of such activity.

## MATERIALS AND METHODS

### Cell culture and viruses.

HEK-293 cells were cultured in Dulbecco’s modified Eagle’s medium (DMEM) supplemented with 10% fetal bovine serum (FBS). HeLa cells were cultured in DMEM supplemented with 8% newborn calf serum (NCS). Both cell lines were maintained at 37°C, 5% CO_2_. Virus stocks were produced in HeLa cells transfected with *in vitro*-transcribed RNA generated from infectious cDNA clones. The following clones were used to generate virus stocks: pT7PV1 for Mahoney strain poliovirus (41), pCVB3-0 for CVB3 Nancy strain (42), and pT7PV1(Δ3′ NCR) for poliovirus Δ3′ NCR (26).

### AUF1 knockdown.

HEK-293 cells were transfected with linearized pSilencer/U6/tetO/shCtrl or pSilencer/U6/tetO/shAUF1 using jetPRIME reagent (Polyplus-transfection) and selected for stable expression using hygromycin B (Calbiochem) as previously described (43).

### Virus infections.

293-shCtrl or -shAUF1 cells were infected with wild-type or Δ3′ NCR poliovirus at a multiplicity of infection (MOI) of 1 or CVB3 at an MOI of 20. Virus was diluted in serum-free DMEM and adsorbed for 30 (poliovirus) or 40 (CVB3) min at room temperature. Following adsorption, cells were overlaid with DMEM supplemented with 10% FBS and incubated at 37°C, 5% CO_2_. Cells and supernatant were harvested at specified time points postinfection, and four freeze-thaw cycles were performed prior to titration by plaque assay on HeLa cells. Virus titers were normalized to cell count and represented as PFU per cell. Values represent the means from triplicate experiments ± standard errors of the means (SEM). Statistical significance was measured by unpaired Student’s *t* test.

### Reverse transcription and quantitative PCR.

293-shCtrl or -shAUF1 cells were infected with poliovirus (MOI of 1) or CVB3 (MOI of 20) as described above. At specified times after infection, cells were harvested in 1 ml TRIzol (Invitrogen) and RNA was extracted according to the manufacturer’s protocol. cDNA was generated from 1 µg total RNA using either oligo(dT)_18_ or virus-specific reverse primers (listed below) and avian myeloblastosis virus (AMV) reverse transcriptase (Life Sciences Advanced Technologies). cDNA was analyzed for viral RNA and glyceraldehyde-3-phosphate dehydrogenase (GAPDH) expression using PowerUp SYBR green master mix (Applied Biosystems) and the 7900HT Fast real-time PCR system (Applied Biosystems). Fold change in viral RNA relative to the 0-h time point for each cell type was calculated using the threshold cycle (ΔΔ*C*_*T*_) method. Virus- or gene-specific primer pairs were as follows: poliovirus, forward, 5′-GTCAATGATCACAACCCGAC-3′, and reverse, 5′-AAGAGGTCTCTATTCCACAT-3′, CVB3, forward, 5′-ACTCTGCAGCGGAACCGACTA-3′, and reverse, 5′-GCTGTATTCAACTTAACAATG-3′; and GAPDH, forward, 5′-GTCCACTGGCGTCTTCAC-3′, and reverse 5′-CTTGAGGCTGTTGTCATACTTC-3′.

### Viral RNA stability measurement.

Cells were infected as described above and incubated for 4 h (poliovirus) or 6 h (CVB3) before vehicle (dimethyl sulfoxide [DMSO]) or 300 µM cordycepin (Tocris Bioscience) was added to the culture medium. Cells were harvested in TRIzol (Invitrogen) at 0, 2, 4, and 6 h after cordycepin addition, and RNA was extracted. RT-qPCR was performed as described above.

### Western blotting.

Proteins were extracted from HEK-293 cells using radioimmunoprecipitation assay (RIPA) buffer (50 mM Tris-HCl, pH 8, 150 mM NaCl, 1% NP-40, 0.5% sodium deoxycholate, 0.1% sodium dodecyl sulfate, protease inhibitor cocktail), and Bio-Rad protein assay dye reagent was used to determine concentration. Equal amounts of protein were resolved by sodium dodecyl sulfate-polyacrylamide gel electrophoresis (SDS-PAGE; 12.5% resolving gel, 5% stacking gel) and transferred to an Immobilon-P membrane (Millipore). Membranes were blocked with 5% nonfat milk in phosphate-buffered saline with Tween 20 (PBST) followed by incubation with rabbit polyclonal anti-AUF1 (1:2,000; Millipore) or rabbit polyclonal antinucleolin (1:1,000; Abcam) antibodies diluted in PBST with 5% bovine serum albumin (BSA). Membranes were washed 3 times with PBST followed by incubation with a 1:4,000 dilution of goat anti-rabbit horseradish peroxidase (HRP)-conjugated IgG heavy and light chain secondary antibody (Bethyl) diluted in PBST with 5% BSA. Membranes were washed 3 times with PBST followed by exposure to ECL Western blotting reagent (Pierce) for chemiluminescent detection of HRP.

### Reporter virus infection and luciferase assay.

*Renilla* luciferase-expressing reporter virus stocks were generated by transfecting RNA transcribed from linearized pT7-R-Luc-PPP (PV-PPP) or p53CB3/T7-RLuc (RLuc-CVB3) plasmids into HeLa cells (44, 45). 293-shCtrl or -shAUF1 cells were plated in 12-well plates (2 × 10^5^ cells/well) and infected with 100 µl of undiluted virus (MOI of <1) as described above. To inhibit viral RNA synthesis, 5 mM guanidine hydrochloride (GuHCl; MP Biomedicals) or 5 mM GuHCl and 5 µg/ml enviroxime (kind gift from Beverly Heinz of Lilly Research Laboratories, Indianapolis, IN) were added during and after adsorption for PV-PPP or RLuc-CVB3, respectively. At 4 h and 8 h postinfection, cells were lysed in *Renilla* luciferase assay lysis buffer and luciferase activity was measured using a *Renilla* luciferase assay system (Promega) and a Sirius luminometer (Berthold Detection System). Luminescence was measured as relative light units per second (RLU/s) normalized to cell count. Data represent the means of triplicate experiments ± standard errors of the means (SEM). Statistical significance was calculated by unpaired Student’s *t* test.

### Immunofluorescence.

HEK-293 cells were plated on glass coverslips and infected with poliovirus as described above. For guanidine hydrochloride (GuHCl; MP Biomedicals) treatment, 5 mM GuHCl was added to both the virus inoculum and growth medium added postadsorption. At specified times after infection, cells were fixed in 3.7% formaldehyde in phosphate-buffered saline (PBS). Fixed cells were washed with PBS and permeabilized in PBS with 0.25% Triton X-100. Cells were washed 3 times with PBS and blocked in 1% BSA in PBS prior to incubation with primary antibody. Cells were incubated with rabbit polyclonal anti-AUF1 (1:100; Millipore) and mouse monoclonal anti-enterovirus capsid (1:500; Dako) antibodies diluted in PBS with 1% BSA followed by 3 washes with PBS. Cells were incubated with goat anti-mouse IgG heavy and light chain DyLight 488- and goat anti-rabbit IgG heavy and light chain DyLight 650-conjugated secondary antibodies (1:400; Bethyl) diluted in PBS with 1% BSA. Following 3 washes with PBS, cells were counterstained with 1 µg/ml 4′,6-diamidino-2-phenylindole (DAPI), and coverslips were mounted on slides with Fluoro-Gel (Electron Microscopy Sciences). Cells were imaged using an LSM700 laser scanning confocal microscope and Zen software (Zeiss).

### *In vitro* transcription, RNA cotransfection, and luciferase assays.

Capped *Renilla* luciferase (RLuc) control RNA was *in vitro* transcribed from the CVB3 bicistronic reporter construct pRstCVB3F (46). pRstCVB3F was linearized with BlpI (New England Biolabs) immediately following the RLuc cistron to prevent transcription of the CVB3 5′ NCR and firefly luciferase cistron. Linearized pRstCVB3F was transcribed in the presence of m^7^G(5′)ppp(5′)G cap analog using the mMessage mMachine T7 transcription kit (Ambion). Uncapped poliovirus or CVB3 5′ NCR-firefly luciferase (FLuc) reporter RNAs were transcribed using the MEGAscript T7 transcription kit (Ambion) from the p5′PVLuc or p5′CVBLuc plasmid linearized with XbaI (47). Cells were seeded in 12-well plates overnight and cotransfected with 0.85 µg 5′ NCR-FLuc and 0.15 µg capped RLuc RNAs using the TransIT mRNA transfection kit (Mirus). To measure the effect of AUF1 on translation of the reporter RNAs during infection, cells were infected with either poliovirus (MOI of 1) or CVB3 (MOI of 20) as described above. Immediately following adsorption, the transfection mixture was added to the culture medium applied to the infected cells. At 4 h and 8 h posttransfection, cells were harvested in passive lysis buffer for 15 min and luciferase activity was measured using the dual-luciferase reporter assay system (Promega) and a Sirius luminometer (Berthold Detection System). Luminescence was measured as relative light units per second (RLU/s) normalized to cell count. Data represent the means from triplicate experiments ± standard errors of the means (SEM). Statistical significance was calculated by unpaired Student’s *t* test.
